# Mineralogical and chemical characterization of Suez Bay surface sediments via multi-analytical techniques

**DOI:** 10.1038/s41598-025-22518-w

**Published:** 2025-10-28

**Authors:** Randa R. Elmorsi, Wael Abdel Wahhab, Khaled S. Abou-El-Sherbini

**Affiliations:** 1 National Institute of Oceanography and Fisheries (NIOF), Cairo, Egypt; 2 Geology Department, National Research Centre, 33 El Bohouth St. (former Tahrir St.), 12622, Dokki, Giza, Egypt; 3 Inorganic Chemistry Department, National Research Centre, 33 El Bohouth St. (former Tahrir St.), 12622, Dokki, Giza, Egypt

**Keywords:** Suez bay, Sediment composition, Mineralogy correlations, Heavy metals, Industrial contamination, Evolutionary ecology, Biogeochemistry, Environmental sciences, Ocean sciences

## Abstract

The mineralogical and chemical characteristics of sediments from Suez Bay (SB) were investigated and correlated with the key water quality parameters. These include polycyclic aromatic hydrocarbons (PAHs), dissolved total phosphorus (DTP), biological oxygen demand (BOD), and total dissolved solids (TDS). Samples were collected from offshore SB sediments, and mass loss at different temperatures (120, 550, and 1000 °C) was analyzed. X-ray fluorescence (XRF) was used to determine the concentrations of 40 elements. X-ray diffraction (XRD) was applied to identify crystalline mineral phases, while Fourier transform infrared (FTIR) spectroscopy was used to characterize functional groups associated with organic matter and carbonate inputs. Major mass losses at 550 and 1000 °C were confirmed to result from the decomposition of organic matter and carbonate minerals, respectively. High enrichment factors and strong inter-element correlations were observed, indicating common sources for specific element groups. A positive correlation was observed between PAHs in sediments and vanadium and nickel, suggesting a petrogenic origin. Significant positive correlations between barium, copper, lead, and zinc likely reflect contamination from ship maintenance activities at the Kazak Hassan West site, south of the SB. In contrast, dissolved total phosphorus (DTP) in the water showed a negative correlation with silica, highlighting their distinct source origins. These findings reveal the distribution patterns of pollutants and their interactions with sediment mineralogy and chemistry, contributing to a clearer understanding of environmental pollution in SB. The results emphasize the influence of proximity to the Suez Canal and human activities on the geochemical behavior of the SB sediments and overlying waters.

**Supplementary Information:**

The online version contains supplementary material available at 10.1038/s41598-025-22518-w.

## Introduction

 Suez Bay (SB) serves as a critical shipping lane and ecosystem, processing over one billion tons of cargo annually while supporting unique Red Sea biodiversity. Its sediments, which have been influenced by both natural and man-made processes, are essential markers of the geochemical dynamics of the area. Determining these sediments’ ecological significance, industrial potential, and environmental impact requires an understanding of their mineralogical and chemical characteristics^1–3^.

Marine sediments play a major role in determining the shore’s geological history and environmental health. Numerous studies have been conducted on their elemental and mineralogical compositions, showing their importance in resource development and environmental monitoring. Marine sediment composition often reflects mixed lithogenic and anthropogenic inputs, integrating contaminants from urban effluents, agricultural runoff, and industrial activities, which require multiple analytical techniques for its investigation^4–6^. Techniques like X ray fluorescence (XRF) and Fourier transform infrared spectroscopy (FTIR) are used to characterize elemental and molecular composition respectively. For example, Ravisankar et al.^7,8^ used XRF to analyze coastal sediments from Tamil Nadu and Andaman Island, India, and found elemental distribution patterns influenced by both natural and man-made factors. Complementing Thermogravimetric Analysis (TGA), X-ray diffraction (XRD), XRF, FTIR spectroscopy provides molecular identification of silicates, carbonates, organic compounds, elemental and minerals composition. By demonstrating FTIR’s ability to differentiate quartz, feldspars, and clay minerals in marine sediments, Hahn et al.^9^ and Hseu et al.^10^ demonstrated how sensitive it is to even the smallest mineralogical changes.

Through the measurement of mass loss during heating, which reflects material composition and stability, TGA offers insights into the thermal behavior of sediments. Bensharada et al.^11^ used TGA to assess organic content and carbonate breakdown in marine sediments. Correlating TGA with mineralogical data facilitates identification of their environmental stability.

Pollutants and heavy metals load of marine sediments are frequently indicators of environmental deterioration originating from urban, agricultural, or industrial sources. El-Moselhy et al.^12^, and Sharaf & Shehata^13^, for example, noted that heavy metal buildup in Suez Gulf and Suez Canal’s, respectively, coastal sediments was a serious problem. On the other hand, the mineral richness of sediments points to possible uses in the water treatment, ceramics, and building sectors.

SB’s sediments are influenced by natural processes and human activities, including the construction and operation of the Suez Canal. Abou-El-Sherbini and Hamed^14^ explored the environmental characteristics of the region. Energy-Dispersive X-ray Spectroscopy (EDX) and XRD analyses revealed that the sediments are primarily composed of four minerals: quartz, calcite, aragonite, and dolomite. The concentrations of carbonate minerals were found to be strongly influenced by land-based activities, leading to the active dissolution of aragonite and calcite and the subsequent preservation of dolomite. However, studies integrating XRF, FTIR, XRD and TGA remain limited.

The contamination of SB surface sediments and aquatic biota with polycyclic aromatic hydrocarbons (PAHs) were studied^3,15,16^. High molecular weight PAHs predominated in the PAH concentrations reported by Abdallah et al.^17^ and Elfadly et al.^18^, which ranged from 621 to 4207 ng g⁻¹ wet mass in fish and from 1667.02 to 2671.27 ng g⁻¹ in sediments. Port operations and oil processing facilities are among the anthropogenic, petrogenic, and biogenic processes that produce these hydrocarbons. The high measured PAH concentrations (1667.02–2671.27.02.27 ng g⁻¹) demonstrates need for ongoing monitoring.

In our recent contributions marine water and sediments of SB are confirmed as important markers of the geochemical processes of the area^3,15^. They represent both natural and man-made causes, such as the construction and upkeep of the Suez Canal. Organic molecules, carbonates, and silicates are among the minerals that comprise sediments^19–21^. The composition of sediments is influenced by industrial discharges, oil processing facilities, and port operations. The presence of heavy metals and PAHs in sediments is a major concern because they can accumulate in aquatic biota and disrupt ecological balance^15^. The regional dataset of Badawy et al.^22^, which reports concentrations of 43 trace elements in Egyptian Red Sea sediments, served as a useful reference. However, because this dataset does not include aluminum and several other elements analyzed, it could not be used directly for EF normalization.

This study provides a comprehensive characterization of surface sediments from the western coast of SB, sampled at a depth of 60 cm, using a multi-analytical approach. What distinguishes this work is the integrated application of TGA, FTIR, XRF, and XRD, statistically-combined with previously published water quality data from the same sites^3,15^—a combination rarely applied simultaneously in sediment studies of this region. This integrated approach offers a holistic understanding of sediment provenance, elemental and mineral composition, and the influence of lithogenic and anthropogenic inputs, thereby providing novel insights into the geochemical and environmental dynamics of the Suez Bay ecosystem.

## Materials and methods

### Site description

SB spans a shallow elliptic extension area from northeast to southwest of the Gulf of Suez towards the Suez Canal southern entrance. According to Hamed et al.^23^, the SB average measurements are 13.2 km in length and 8.8 km in width, with a surface area of 77.13 km² and a mean depth of 10 m. It features a 12-meter-deep waterway on its northeastern side that connects to Suez and a 20-meter-deep navigational canal on its southeast side that connected to the GS^1–3^.

**Fig. 1:**
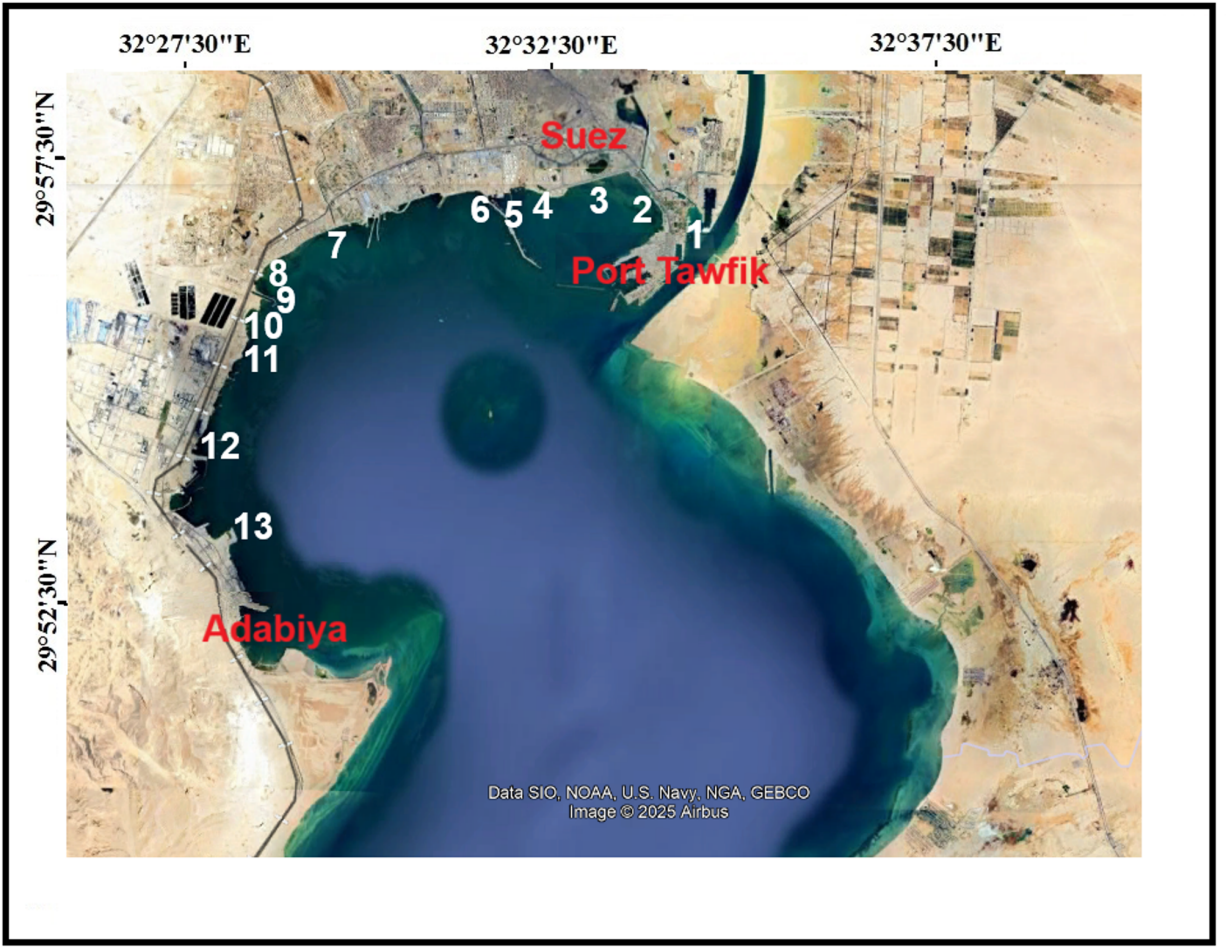
Sampling locations from 13 sites along the western coast of Suez Bay. Map was generated using Google Earth Pro. Data SIO, NOAA, U.S. Navy, NGA, GEBCO. Image © 2025 Airbus.

The study area, SB, is located in the northwestern part of the Gulf of Suez, in the Red Sea (Fig. 1). SB has two important ports, Adabiyah and Tawfik in the southern and norther ends of the west side, which receive discharges from anthropogenic, commercial and industrial activities that can affect water quality. The western side of SB also receives sewage and waste both from the city of Suez and from ships awaiting transport through the Suez Canal. In addition, it receives waste from the industrial complex south of Suez, including oil refineries, a fertilizer plant, power stations and other industries. All types of waste coming from different sources are directly or indirectly discharged with or without treatment into SB. This waste contains a wide variety of chemical residues, including aromatic derivatives^24^. Table 1 summarizes sample locations.

**Table 1:** Sample locations and their GPS coordinates.

Number	Location	Latitude *N*	Longitude E
1	Naval base	29ᵒ 57′ 06″	32ᵒ 34′ 31″
2	Port Tawfik	29ᵒ 57′ 16″	32ᵒ 33′ 50″
3	Courniche	29ᵒ 57′ 27″	32ᵒ 33′ 08″
4	Salakhana	29ᵒ 57′ 17″	32ᵒ 32′ 22″
5	Pilgrim Village	29ᵒ 57′ 04″	32ᵒ 32′ 00″
6	Nasr Petroleum Company	29ᵒ 57′ 12″	32ᵒ 31′ 38″
7	Cabanon drain	29° 56’ 44”	32°29’36″
8	Fertilizers and Misr-Iran Companies	29°56’15″	32° 28’ 41″
9	Attaqa Electrical Station	29° 56’ 7″	32° 28’ 37″
10	NIOF	29° 55’ 32″	32° 28’ 32″
11	Kazak Hassan	29° 55’ 14″	32° 28’ 21″
12	Attaka	29ᵒ 54′ 06″	32ᵒ 28′ 02″
13	Adabiya	29ᵒ 53′ 02″	32ᵒ 28′ 21″

### Sampling procedure

Sediment samples were collected from 13 different sites on the west coast of SB (Fig. 1). Offshore sediments (5–6 grabs within a 5 m diameter area) from each site were mixed well and then poured into pre-cleaned wide-mouth glass bottles. Samples were stored at 4 °C and handled immediately upon return to the laboratory according to standard sediment protocols^25^.

### Analytical techniques

Sediment samples (100–200 g air dried) were homogenized and milled to 200 mesh. Fourier Transform Infrared (FTIR) spectra were recorded using a ThermoFischer Scientific and a Bruker ALPHA-FTIR-Spectrometer with a platinum attenuated total reflectance (ATR) module, covering a range from 400 to 4000 cm^−1^.

Thermogravimetric analysis (TGA) was performed on 5 g of each air-dried sediment sample, placed in a porcelain crucible and heated in a muffle furnace under ambient air at 120 °C, 550 °C, and 1000 °C for three hours. After cooling to room temperature in a desiccator, the residual mass was recorded, and mass loss percentages were calculated relative to the initial sample weight.

X-ray diffraction (XRD) measurements were conducted using a BRUKER D8 diffractometer with a Cu_kα_ radiation and a Ni filter at constant voltage 40 kV, and 40 mA.

Chemical analyses of major oxides (wt%) and some trace elements in the sediments were performed using an X-ray fluorescence (XRF) spectrometer (Axios, WD-XRF Spectrometer, PANalytical, 2005, Netherlands).

### Enrichment factor (EF)

The EF was calculated according to Eq. (1)^26^:

1 $$\:\text{E}\text{F}=\:\frac{{(\text{X}}_{\text{s}}^{\text{i}}/{\text{X}}_{\text{s}}^{\text{A}\text{l}})}{{(\text{X}}_{\text{E}\text{C}}^{\text{i}}/{\text{X}}_{\text{E}\text{C}}^{\text{A}\text{l}})}$$

$\:{\text{X}}_{\text{s}}^{\text{i}}$ is the concentration (µg g^−1^) of a heavy metal in the sediment sample, $\:{\text{X}}_{\text{s}}^{\text{A}\text{l}}$ is the concentration (µg g^−1^) of aluminum as an immobile metal in the sample and $\:{(\text{X}}_{\text{E}\text{C}}^{\text{i}}/{\text{X}}_{\text{E}\text{C}}^{\text{A}\text{l}})$ is the natural abundance ratio of the metal ion (i) in the earth’s crust to the immobile reference normalizing element Al^27^. The natural abundances of the studied elements were derived from the widely cited upper continental crust values in the literature^28^, due to the absence of site-specific baseline data for Suez Bay sediments.

### Statistical analysis

For statistical analysis, a bivariate two-tailed Pearson correlation test was used to evaluate the significant difference in the concentrations of metals at different study sites and previously reported water quality parameters at the same exact sites^15,29^. A probability of a level of 0.05 or less was considered significant.

## Results

### Thermal decomposition behavior

**Fig. 2:**
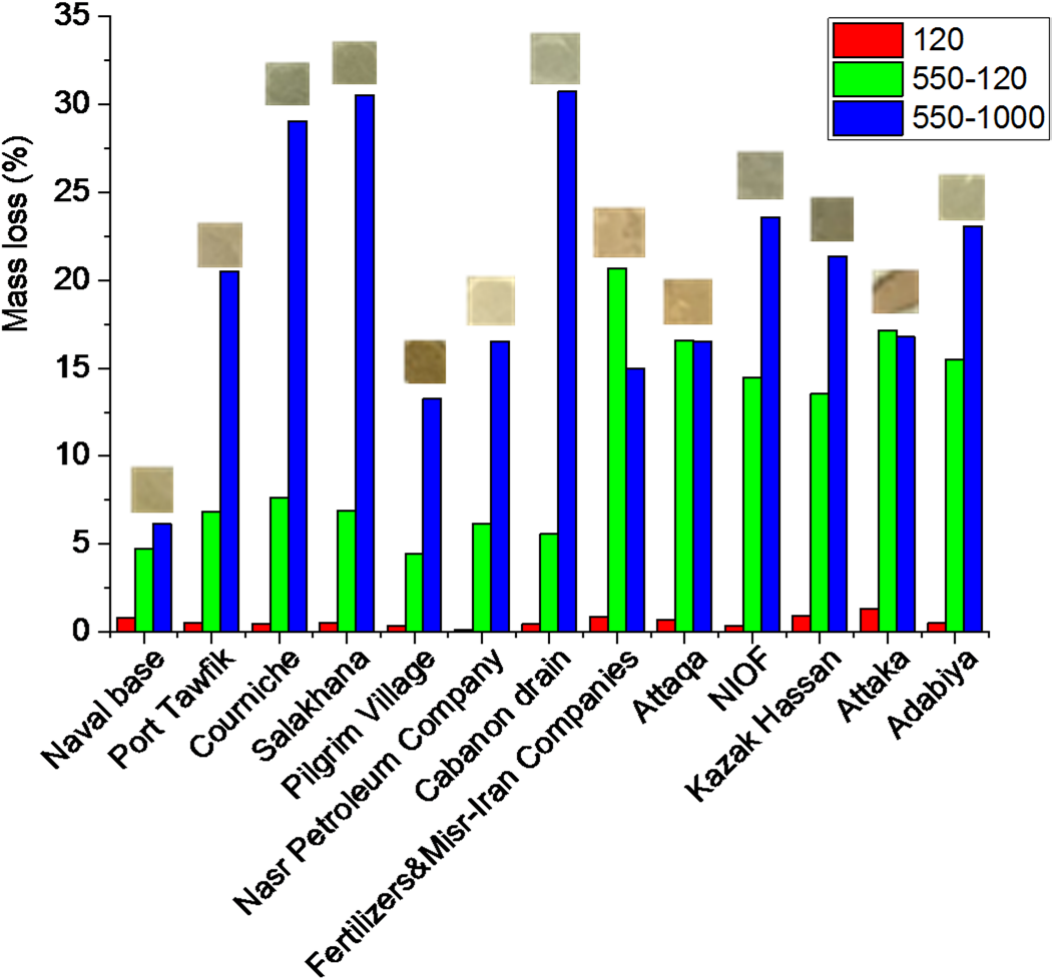
Mass loss (%) after thermal treatment at 120–1000 °C for air-dried sediments. Residues of the thermal treatment processes are shown above.

Figure 2 present the mass loss of dried sediments in the temperature range from room temperature to 1000 °C. In the range from room temperature to 120 °C, the mass loss varied from 0.13% at the Nasr Petroleum Company to 1.35% at Attaka, with an average value of 0.61%. From room temperature to 550 °C, the mass loss ranged from 4.77% at the Pilgrim Village to 21.52% at Fertilizers & Misr-Iran Companies, with an average value of 11.40%.

For the entire range from room temperature to 1000 °C, the mass loss ranged from 11.7% at the Naval Base to 39.09% at Adabiya, with an average value of 31.66%. Between 120 and 550 °C, the mass loss was between 4.43% at the Pilgrim Village and 20.68% at Fertilizers & Misr-Iran Companies, with an average of 10.79%. Finally, in the range from 550 to 1000 °C, the mass loss ranged from 6.16% at the Naval Base to 30.79% at the Cabanon Drain, with an average value of 20.26%. These trends are further illustrated in Fig. 2, which also shows the picture of the final residues that ranged in color from pale gray, orange, brown to dark gray, indicating varied compositions. Table S1 details TGA mass loss results.

### Molecular vibrations

**Fig. 3:**
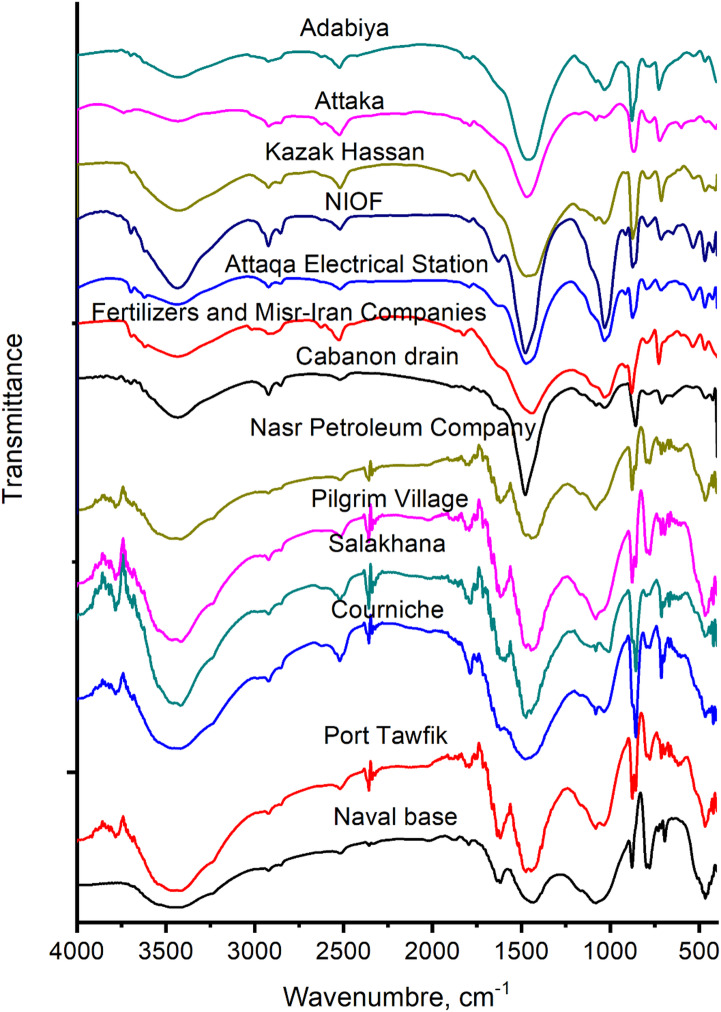
FTIR of air-dried sediments.

Figure 3 shows the FTIR spectra of the dried sea sediments collected from various locations in the western side of SB. Broad peaks around 3400 cm⁻¹ typically correspond to O-H stretching. Peaks in the range 3000–2800 cm^−1^ suggest C-H stretching. Peaks around 1800 cm^−1^ may correspond to C = O stretching while those at approximately 1624 cm⁻¹ are due to O-H bending^30^. Strong peaks in the ranges 1500–1400 and 1100–1000 cm⁻¹ indicate carbonate and silicate minerals, respectively^31,32^. Duplicate peaks in the ranges 900–830 and 830–750 cm⁻¹ indicate carbonate and silicate minerals. The assignment details of the FTIR peaks are given in Table S2 for selected samples.

The Attaka and Adabiya sediments showed the weakest intensities of silicate mineral bands, whereas the Naval Base site exhibited the strongest intensity. The sites extending from the Cabanon drain to Attaka displayed appreciable C–H stretching bands, while the weakest intensities of these bands were observed in the sediments from the Naval Base and the Nasr Petroleum Company area. The strongest carbonate bands were detected in the Courniche, Salakhana, and Cabanon drain sediments, whereas the weakest carbonate band intensities were found in the Naval Base sediment.

### Elemental composition

**Fig. 4:**
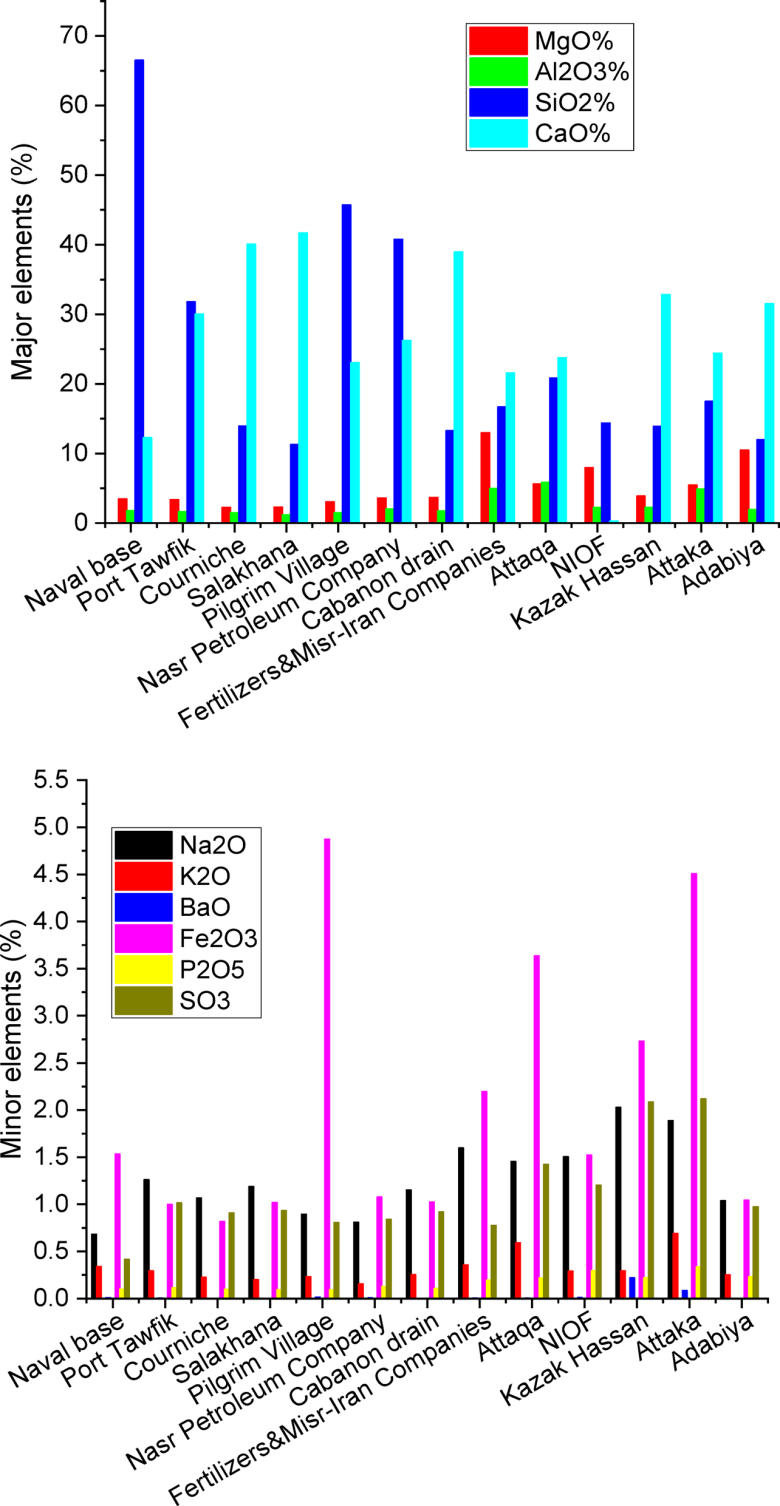

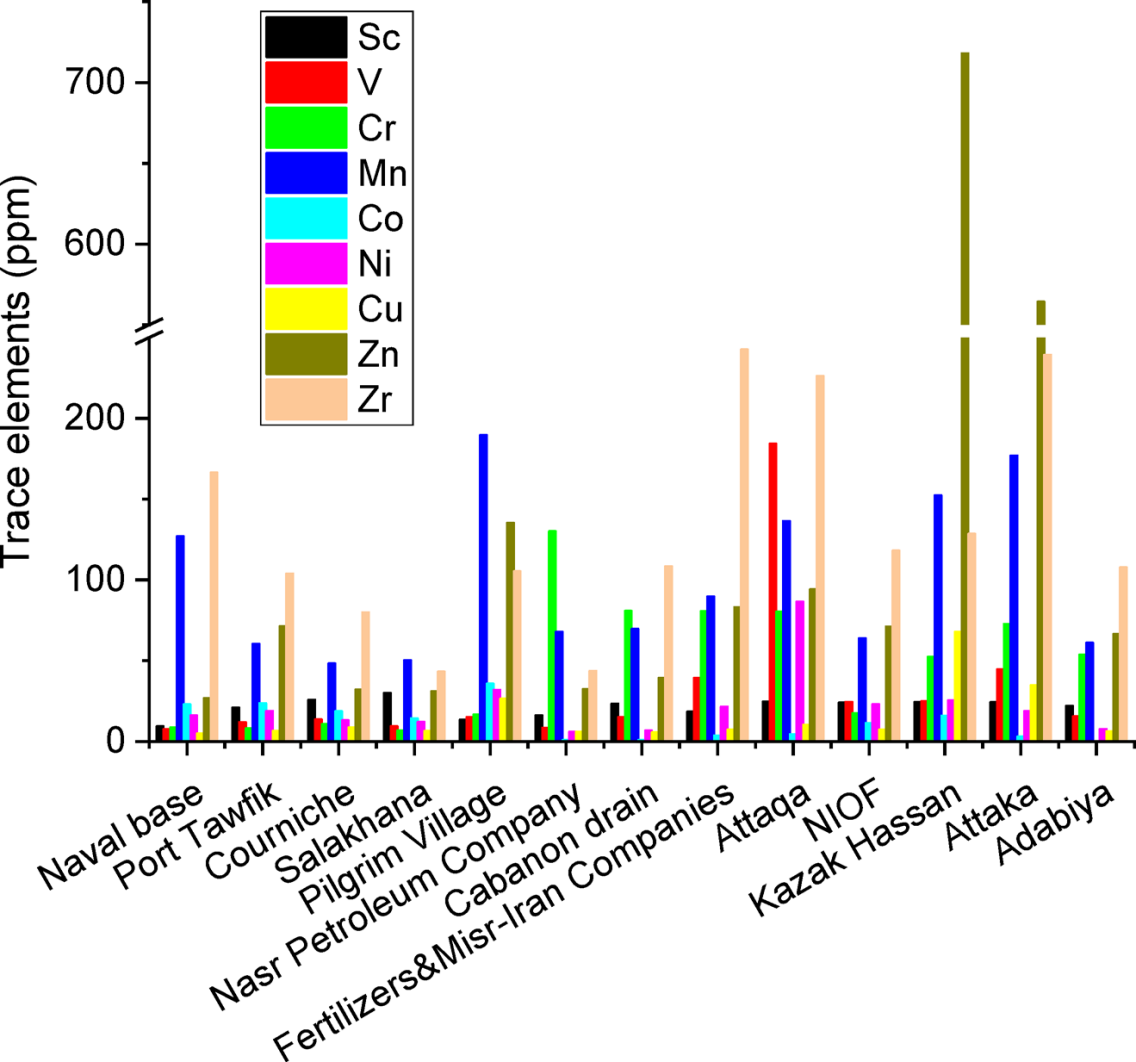
XRF analysis of air-dried sediments.

Figure 4 displays the XRF analysis of the air-dried sediments for selected key elements, while the remaining elements are presented in Supplementary Figure S1. The most abundant major elements were Ca followed by Si then Mg, and Al. The naval base was characterized by the highest silica content (66.52%) followed by the pilgrim village (45.70%) and the Nasr Petroleum Company (40.78%). The pilgrim village was characterized by the lowest silica content (11.30%) followed by the Adabiya Port (11.99%) and the Cabanon drain (13.28%). The highest calcium oxide content was observed at the pilgrim village (41.70%) followed by the Salakhana (40.07%) and the Cabanon drain (38.95%). The highest calcium oxide content was observed at the pilgrim village (41.70%) followed by the Salakhana (40.07%) and the Cabanon drain (38.95%). The lowest calcium oxide content was observed at the NIOF (0.30%) followed by the naval base (12.28%) and the Fertilizers & Misr-Iran Companies (21.58%).

The most abundant minor elements were Fe followed by Na then Cl, S, Ti, Sr, K and P with average oxide (except Cl) concentrations of 2.08, 1.27, 1.27, 1.11, 0.40, 0.36, 0.32 and 0.17%, respectively. The highest iron concentration was obtained at the pilgrim village (4.85%), the Attaka Port (4.51%) and Attaqa (3.64%).

The most abundant trace elements were Zn followed by Zr then Mn and W with average concentrations 151.22, 131.87, 99.54 and 80.63 mg kg^−1^, respectively. The highest zinc concentration were obtained at the Kazak Hassan (718.2 mg kg^−1^), the Attaka Port (564.30 mg kg^−1^) and at the pilgrim village (135.40 mg kg^−1^).

The most abundant f-block elements were Ce and Th with average concentrations 13.18 and 8.69 mg kg^−1^, respectively. The highest Ce concentration was obtained at the NIOF (36.00 mg kg^−1^). Detailed data are provided in Supplementary Files S3 (Excel spreadsheet) and S4 (raw data images).

### Crystalline phases and mineral composition

**Fig. 5:**
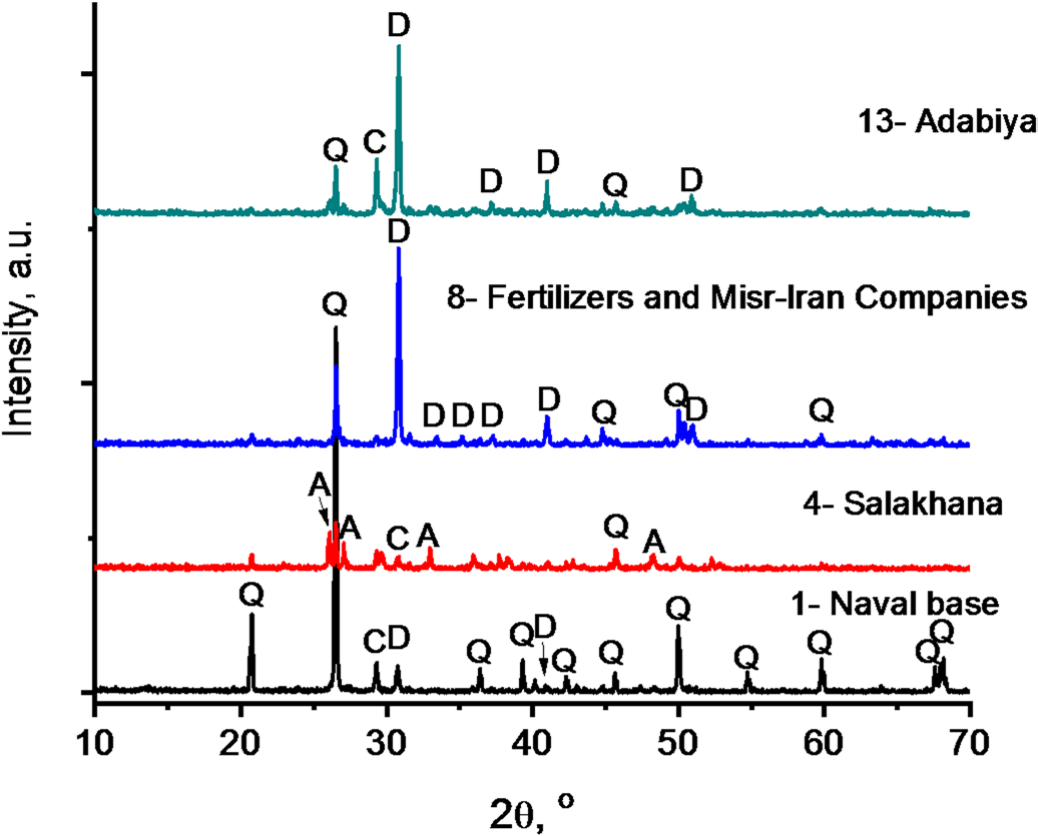
XRD of air-dried sediments. Q, D, C, and A denote quartz (96–900-9667), dolomite (96–900-3525), calcite (96–901-6707) and aragonite (96–901-4888), respectively.

Figure 5 represents the XRD measurements of selected air-dried sediment samples. The dominant mineral phases identified include quartz (SiO_2_, PDF no. 33–1161), calcite (CaCO_3_, PDF no. 5–586), aragonite (CaCO_3_, PDF no. 41–1475) and dolomite (CaMg(CO_3_)_2_, PDF no. 36–426). The sediment sample from the Naval Base is primarily composed of well-crystalline quartz, with dolomite and calcite appearing as minor constituents (relative intensities = 7.31 and 8.34%, respectively). In contrast, the Salakhana sample exhibits major diffraction peaks corresponding to quartz, aragonite, and calcite, indicating their order of abundance (relative intensities = 100, 79.62 and 39.09%, respectively). Also, at the Fertilizers & Misr-Iran Companies and Adabiya sites, dolomite emerges as the predominant phase, accompanied by minor amounts of quartz (relative intensities = 41.31%) at the Fertilizers & Misr-Iran Companies and quartz and calcite at Adabiya (relative intensities = 33.69 and 29.04%, respectively). These variations reflect differences in sediment composition likely influenced by local geological and anthropogenic factors.

### Enrichment study

**Fig. 6:**
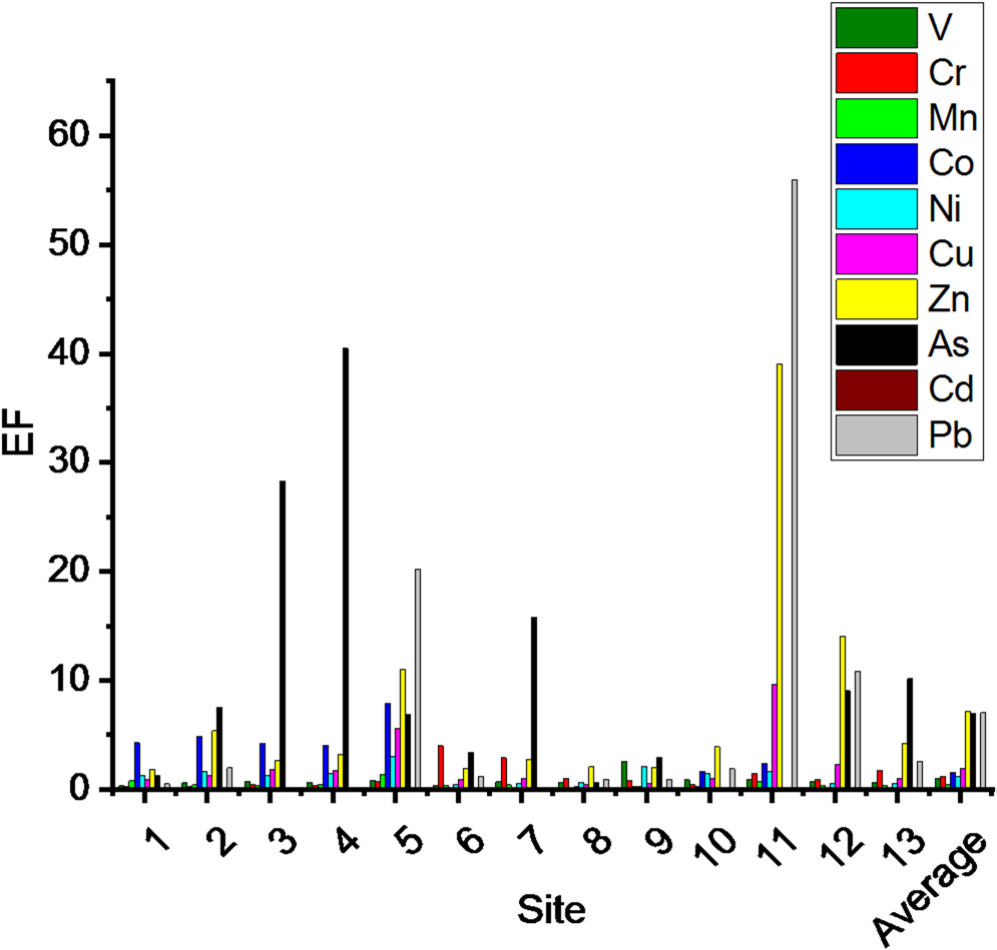
Enrichment factors of some metal(oid)s in the sediments of Suez Bay.

In the absence of site-specific baseline concentrations for SB sediments, particularly for aluminum as the normalizing element, globally accepted upper continental crust values were used. Figure 6 shows the EFs of the some studied metal(oid)s; V, Cr, Mn, Co, Ni, Cu, Zn, As, Cd and Pb in sediments, compared with the normalizing element (aluminum), that was chosen as an immobile metal. All metals were more abundant than Al, except Mn and Cd with average metal(oid) EFs of 0.01–7.18 across the SB sites. The EFs increased in the following order: Cd < Mn < V < Cr < Ni < Co < Cu < As < Pb < Zn. Additionally, the average EFs for the site were within the range of 1.15–11.16 and increased in the following order: 1 < 8 < 6 < 9 < 10 < 2 < 7 < 13 < 3 < 12 < 4 < 5 < 11. The maximum EF was 55.93 for Pb at site (11), whereas the lowest EF was 0 for Cd at sites 1, 3, 4, 6, 7, 8, 12 and 13. Detailed enrichment factors are present in S3. However, while EF values provide a useful indication of potential anthropogenic enrichment, their interpretation should be made with caution.

### Statistical analysis

A statistical evaluation was conducted to examine the relationships between mass loss values across thermal treatment ranges (room temperature to 120 °C, 120–550 °C, and 550–1000 °C), XRF elemental compositions, and the published water quality parameters from previous study at the same specific locations^15,29^. The results are detailed in Supplementary File S5 (Excel spreadsheet). Strong positive correlations (r = > 0.9) were observed between the groups of parameters Al with Ga, Rb, and Nb; Na with Cl; mass loss at 550–1000 °C with Ca; Fe with Mn; V with Ni and Br; Co with W; Cu with Zn, Ba and Pb; Ga with Al, Rb and Nb; Zn with Cu, Ba and Pb; Rb with Al, Ga, Y, Zr and, Nb; Sr with Th; Y with Rb, Zr and Nb; Zr with Y, Rb and Nb; and Ba with Cu, Zn, and Pb.

Good positive correlations (*r* = 0.7 < 0.9) were observed for the variants groups: I with Sc and Ce; La with V and Sm; Br with Rb, Y, Zr, and Nb; As with Sr and Th; Y with Hf; Mo with Ba and Pb; Ag with Cd; Ni with Br, Sm and PAHs; Br with Al, K, Ni, Ga, Y, Cl, Rb, Zr, mass loss at 550–1000 °C and PAHs; Cu with S, Ti, and Mo; Zn with mass loss at 120 °C, Na, S, Ti, Cl and Mo; Ga with mass loss at 120–550 °C, K, V, Br, Y and Zr; mass loss at 120–550 °C with U, Nb, Zr, Y, Rb, Br, Ga, Cl, Al, Na, Mg and P; Mass loss at 120 °C with K, Ti, Zn and Zr; Mass loss at 550–1000 °C with Sr, Th and Sc; Na with P, S, Ti and Zn; Mg with U; Al with P, Cl, V, Br, Y and Zr; P with S; K with Ga, Br, Rb, Y, Zr and Nb; S with Cl, Ti, Cu, Zn, Mo, Ba and Pb; Ca with Sc, Sr and Th; Cl with Ti, Zn and Br; Ti with Mo, Ba and Pb; Sc with I; V with Nb and La; PAHs with Sc, V, Ni and Br; BOD with Bi and As; DTP with pH and TDS with S.

Good negative correlations (*r*=−0.7>−0.9) were observed between the groups of variants DTP with Si; TDS with Bi; mass loss at 550–1000 °C with Si and Hf; Si with Ca and Sc; Ca with Y and Hf; and Cr with W and Bi.

## Discussion

Adabiyah and Tawfik Ports (sites 13 and 2, respectively) facilitate commercial and industrial operations, making SB a center of economic activity. However, there are some environmental concerns as a result of these activities as well as industrial and urban emissions. The city of Suez (its major drain is at site 7 while smaller drains are present elsewhere such as near Salakhana at site 4 and Pilgrim village at site 5), ships, and industrial complexes such as power plants, fertilizer factories (site 8), and oil refineries all contribute untreated trash to the bay.

### Mineralogical composition and geochemical processes

The mineralogical profile of SB sediments reflects an interplay between natural lithogenic inputs and anthropogenic pressures. Quartz exists across all sites, consistent with detrital inputs from the surrounding desert, while carbonate minerals (calcite, aragonite, and dolomite) are largely derived from biogenic sources such as shells and skeletal debris and from lithogenic sources such as Attaqa Mountain, which is formed from dolomite^33^. However, the selective dissolution of aragonite and calcite, coupled with localized dolomite precipitation, indicates shifts in geochemical conditions likely driven by industrial and urban discharges, which was attributed to warm discharges from the Attaqa power station^14^. Similar dissolution–precipitation dynamics have been reported in other impacted coastal systems, where the fresh water inflows reduce pH and decreases salinity that promote carbonate dissolution and secondary mineral formation^34^.

The occurrence of aragonite only at site 3 highlights a localized geochemical environment favorable for aragonite stability and its breakdown in the other sites. This general SB pattern likely reflects the influence of low pH and elevated temperature conditions associated with untreated domestic effluents^14^. Comparable observations have been documented in semi-enclosed bays subject to similar anthropogenic stress caused by the discharge of inadequately treated industrial effluents^35^.

Thermal and spectroscopic analyses further support these geochemical interpretations. The studied thermal ranges 0–120, 120–550, 550–1000 °C were selected as they mainly attributed to moisture loss, decomposition of organic matter and carbonates, respectively^36^. For example, the mass loss values in the range 120–550 °C mainly represent the thermal decomposition of organic content^37–39^. Also, dehydroxylation of the present clay minerals may occur in this stage^40,41^. The presence of its highest amount at site 8 may be attributed to imperfectly-treated effluents from the fertilizers company while the Cabanon drain may partly share the organic load due to the anticlockwise circulation of water current in SB^3,14,15^. This may also explain the gradual decrease in the values of this mass loss stage from site 8 to 13. The strong mass loss above 550 °C detected by TGA corresponds to carbonate breakdown^42^, particularly of aragonite, calcite, and dolomite, and aligns well with the high CaO and MgO contents measured by XRF at sites with abundant shells and eutrophication state^3^; 3 (42.33%), 4 (44.00%) and 7 (43.64%). Likewise, the FTIR spectra showing strong carbonate and silicate bands across most sites corroborate the combined lithogenic and biogenic origins of these sediments. Collectively, these results indicate that while the natural sediment supply defines the mineral framework, anthropogenic affects the geochemical pathways governing carbonate stability and mineral transformations in SB.

### Organic matter and potential ecological risks

Organic matter in Suez Bay sediments originates from a combination of natural inputs (e.g., planktonic productivity, detrital matter) and anthropogenic sources such as untreated sewage, industrial effluents, and petroleum-related discharges^15,24,43,44^. This dual origin is supported by TGA mass losses in the 120–550 °C range, mainly attributed to organic matter decomposition^37–39^, and by FTIR peaks corresponding to C–H and C = O stretching vibrations, which are characteristic of humic substances and petroleum hydrocarbons^45^. Similar diagnostic patterns have been reported in other industrialized coastal zones^46^.

Spatial trends reveal that sites near the fertilizer complex and the Cabanon drain exhibit the highest organic loads, reflecting the influence of poorly treated industrial and urban discharges. The anticlockwise circulation of water currents within the bay likely facilitates the transport and accumulation of these organic contaminants in localized zones^3,14,15^. In contrast, the Naval Base area and the Nasr Petroleum Company site display the lowest organic signatures, indicating relatively limited anthropogenic input in these sectors.

Of particular concern is the observed good correlations between of elevated TGA-derived organic matter with heavy metals U, Nb, Zr, Y, and Ga. This association suggests the potential formation of metal–organic complexes, which can increase the mobility and bioavailability of these contaminants in the marine environment. Such interactions have been shown to pose significant ecological risks, including bioaccumulation in micro-organisms and subsequent transfer through the food chain^47^. These correlations highlight the need for targeted risk assessments to evaluate the implications for marine biodiversity and seafood safety.

### Enrichment and Spatio-Temporal trends of selected heavy metals in sediment quality

EFs of < 1, <3, 3–5, 5–10, 10–25, 25–50 or > 50 indicate no, minor, moderate, moderate to severe, severe, very severe, or extremely severe enrichment, respectively^48^. Based on these thresholds, most sites in SB exhibit minor to moderate enrichment with the studied elements V, Cr, Mn, Co, Ni, Cu, Zn, As, Cd and Pb. Sites near urban drains (e.g., Pilgrim Village and Salakhana) exhibited moderate to severe enrichment of As, indicating untreated domestic effluents.

Ports, ship maintenance areas, and industrial complexes, significantly contribute to pollutant loads of the bay, consistent with previous observations for this region and other semi-enclosed marine systems^22,23,43,49^. Elevated enrichment factors (EFs) of Zn, Pb, and As—notably at Kazak Hassan, Attaka Port, and Salakhana—point to direct industrial and ship-related discharges. For example, the extremely high EF values of Pb (59.9) and Zn (39.0) - largely linked to untreated industrial discharges and ship maintenance activities^49^ - at Kazak Hassan align with their known association with antifouling paints and other marine coatings^50,51^. These findings are supported by strong positive correlations between Cu, Zn, Ba, and Pb, reflecting common anthropogenic sources, as well as reports of similar contamination pathways in SB^14,49^ and for other ports such as Trieste **(**Adriatic Sea)^26^.

The chemical and mineralogical characteristics of SB sediments clearly reflect the influence of anthropogenic activities. Spatial trends show localized inputs: urban drains affecting areas from Courniche to Pilgrim Village and shipping activities impacting Kazak Hassan and Attaka Port. Although several hot spots remain concerning, generally lower concentrations of the metals compared to earlier studies^22,24,52–54^ suggest early benefits of recent environmental management initiatives. A comparison of the present findings with reported datasets (Table 2) reveals both improvements and persistent challenges in SB sediment quality. Overall, concentrations of several trace metals—particularly Pb, Cu, and As—are lower than those levels documented in other impacted coastal systems such as the Tyrrhenian Sea (Italy)^55^, the Bay of Bengal (India)^56^, and the Trieste area of the Adriatic Sea^26^.

Hydrodynamic conditions further influence these patterns: the anticlockwise circulation of water within the bay promotes the redistribution and accumulation of contaminants from the urban drains near sites 3 and 4 to the south^3,14,15^. These findings highlight the importance of integrating spatial dynamics into environmental management strategies, ensuring that monitoring and remediation efforts are directed toward the most vulnerable areas.

### Comprehensive inter-element relationships in SB sediments

Correlation analysis provides complementary insights into geochemical processes and pollutant sources in SB sediments. Several strong associations (*r* > 0.9) were detected, notably between Na–Cl, Fe–Mn, Al–Ga–Rb–Nb, Cu–Zn–Ba–Pb, and mass loss at 550–1000 °C with Ca. At higher temperatures (550–1000 °C), strong correlations with Ca, Sr, and Th confirmed the link to carbonate decomposition. These patterns are consistent with seawater salinity, common lithogenic controls, and anthropogenic inputs from ship maintenance and industrial activities mostly highlighted in Sect. 4.1–4.3. The strong clustering of Al with Ga, Rb, and Nb further suggests contributions from external industrial sources such as coal ash^57^, which are likely distinct from the effluents from Attaqa Power Station.

Beyond these primary relationships, several good correlations (0.7 < *r* < 0.9) linked metals and water-quality parameters, reinforcing the interaction between geochemistry and pollution pathways. For example, PAHs correlated positively with V, Ni, and Br, supporting earlier conclusions of a petrogenic origin^58^. A clos conclusion was reported by the analysis of the phenanthrene/anthracene ratios in the SB, which were mostly > 5 indicating a petrogenic origin of PAHs^15^. Similarly, TDS correlated with S, likely due to the common ion effect, whereas detergent-related phosphates (DTP) showed negative correlations with silica, pointing to distinct anthropogenic pathways. Negative correlations, such as those between Si and Ca, or between Ca and Y–Hf, indicate localized carbonate dissolution and trace element redistribution in sandy sediments^20,21^.

Taken together, these findings extend the mineralogical and enrichment patterns described in Sect. 4.1–4.3 by revealing integrated element groupings (e.g., Cu–Zn–Ba–Pb, Al–Ga–Rb–Nb, PAHs–V–Ni) and their connections to geogenic, industrial, and petroleum-related sources. This holistic view highlights the importance of combining multivariate geochemical relationships with pollutant monitoring to guide effective environmental management in SB.

**Table 2:** Comparison concentrations (mg kg^−1^) of some heavy metals in sediments of SB with reported results.

Site	V	Cr	Mn	Co	Ni	Cu	Zn	As	Cd	Pb
This work, lowest-highest (mean)	0.33–2.56 (0.81)	0.31–4.01 (1.20)	0.20–1.41 (0.50)	0.03–7.92 (2.33)	0.42–3.03 (1.27)	0.48–9.61 (2.19)	1.85–39.09 (7.24)	ND-40.47 (9.73)	ND-0.08 (0.01)	ND-55.94 (7.47)
SB^24^	-	1.56–101.3 (44.69)	-	-	7.55–312.00 (35.75)	3.31–85.07 (18.10)	9.17–451.1 (89.19)	-	1.04–2.29 (1.67)	6.22–67.99 (21.95)
Red Sea^22^	6–126 (29.8)	1–156 (53.8)	30–1580 (291.9)	0.3–8.1 (4.8)	1.1–45 (15.4)	0.8–14.8 (7.7)	2.6–58.9 (27.6)	0.5–18.5 (6.3)	-	1–8.8.8 (4.9)
Suez Gulf^52^	-	-	70.27	-	-	15.25	-	-	1.31	17.96
Suez Gulf^54^	-	-	67.61	11.77	17.26	3.70	24.25	-	2.68	22.96
Tyrrhenian sea^55^	-	39.38–111.56.38.56	-	-	13.35–88.64	2.84–38.72	-	20.07–85.26	0.01–0.28	1.69–147.12.69.12
Bay of Bengal^56^	-	16.48–74.70	70.27–346.22.27.22	1.80–9.02	5.73–40.53	2.01–53.78	4.73–53.54	2.09–28.18	0.20–21.76.20.76	1.37–17.54
Trieste^26^	25.0- 196 (128)	51.3–215 (145)	181–742 (486)	4.86–19.5 (13.3)	19.8–116 (74.3)	4.76–140 (31.9)	28.4–434 (131)	6.54–39.6 (15.9)	0.04–0.90 (0.15)	7.43–223 (54.4)

## Conclusion

This study provides a comprehensive geochemical and mineralogical characterization of Suez Bay surface sediments using an integrated suite of TGA, XRF, FTIR, and XRD analyses. The findings demonstrate a complex interplay between lithogenic and biogenic inputs and strong anthropogenic influences, particularly from industrial discharges and maritime activities.

FTIR and XRD confirmed the coexistence of silicates, carbonates, and organic matter, while the presence of a distinct C-H and C = O absorption bands indicated organic degradation products. The detection of aragonite exclusively at Site 3 while it disappears in other sites points to delocalized pollution stress in most of SB. Thermogravimetric mass-loss profiles further reflected contributions from moisture, organic matter, and carbonate breakdown, consistent with mixed natural and anthropogenic sources.

XRF analysis highlighted spatial variability in 40 elements, with hotspots of potentially toxic metals (As, Cu, Pb) near effluent outlets and port facilities. Enrichment factor (EF) calculations confirmed minor to moderate enrichment at most sites but revealed severe to extreme contamination at specific hotspots (e.g., Pb up to EF 59.9 at Kazak Hassan from ship-painting activities). These results underscore the ecological risks of bioaccumulation and sediment toxicity.

Correlation analysis expanded the interpretive framework by revealing broader inter-element relationships. Strong associations (e.g., PAHs–V–Ni, Cu–Zn–Ba–Pb, Al–Ga–Rb–Nb, Si↔Ca) linked pollutants to petrogenic inputs, shipyard maintenance, coal ash contributions, and carbonate dissolution processes. Thermogravimetric–elemental linkages (e.g., mass loss at 120–550 °C with Na, Mg, Al, P, and Cl) pointed to co-mobilization of lithogenic and anthropogenic fractions, raising concerns about potential transfer of trace elements into the food chain.

Altogether, the integration of mineralogical, chemical, and statistical evidence provides a robust framework for identifying contamination hotspots and apportioning sources in semi-enclosed marine systems. The results highlight the promising attitude of pollution management of lowering risk levels particular but also show vulnerability of SB to industrial and maritime pollution and reinforce the need for stricter wastewater controls, improved regulatory enforcement, and sustained monitoring programs to safeguard its ecological integrity.

## Supplementary Information

Below is the link to the electronic supplementary material.

Supplementary Material 1

Supplementary Material 2

Supplementary Material 3

Supplementary Material 4

Supplementary Material 5

Supplementary Material 6

Supplementary Material 7

Supplementary Material 8

## Data Availability

All data generated or analyzed during this study are included in this published article and its Supplementary Information files (Supplementary Figure S1; Tables S1&S2; Excel files S3 and S5; and raw data as S4, S6 and S7). The X-ray diffraction (XRD) datasets generated and/or analyzed in this study are available in the Mendeley Data repository: Abou-El-Sherbini, Khaled (2025), “XRD data of sediments from Suez Bay – Egypt”, Mendeley Data, V1, [https://doi.org/10.17632/98bmbkmb2s.1](https:/doi.org/10.17632/98bmbkmb2s.1).For further information or data access requests, please contact Prof. Dr. Khaled Abou-El-Sherbini via email: ks.elsaid@nrc.sci.eg.
