# Supplementary material for: Mineralogical and chemical characterization of Suez Bay surface sediments via multi-analytical techniques
Source: Sci Rep. 2025 Oct 28;15:37729. doi: 10.1038/s41598-025-22518-w (PMC12568952; doi:10.1038/s41598-025-22518-w)
Supplement: Supplementary file 2 — Supplementary Material 2 [file 41598_2025_22518_MOESM2_ESM.docx]

**Mineralogical and Chemical Characterization of Suez Bay Surface Sediments via Multi-Analytical Techniques**

Randa R. Elmorsi^a^, Wael Abdel Wahhab^b^, Khaled S. Abou-El-Sherbini^,c^

*^a^ National Institute of Oceanography and Fisheries (NIOF), Egypt.*

*^b^ Geology Department, National Research Centre, 33 El Bohouth St. (former Tahrir St.), 12622, Dokki, Giza, Egypt.*

*^c^ Inorganic Chemistry Department, National Research Centre, 33 El Bohouth St. (former Tahrir St.), 12622, Dokki, Giza, Egypt.*

Table S1 TGA mass loss (%) of dried sediments in the temperature range from room temperature to 1000 °C.

| Location | | Mass loss, % | | | | |
| --- | --- | --- | --- | --- | --- | --- |
|  |  | 25-120°C | 25-550°C | 25-1000°C | 120-550°C | 550-1000°C |
| 1 | Naval base | 0.79 | 5.54 | 11.7 | 4.75 | 6.16 |
| 2 | Port Tawfik | 0.53 | 7.36 | 27.88 | 6.83 | 20.52 |
| 3 | Courniche | 0.49 | 8.13 | 37.21 | 7.64 | 29.08 |
| 4 | Salakhana | 0.52 | 7.43 | 37.98 | 6.91 | 30.55 |
| 5 | Pilgrim Village | 0.34 | 4.77 | 18.06 | 4.43 | 13.29 |
| 6 | Nasr Petroleum Company | 0.13 | 6.28 | 22.82 | 6.15 | 16.54 |
| 7 | Cabanon drain | 0.46 | 6.04 | 36.83 | 5.58 | 30.79 |
| 8 | Fertilizers & Misr-Iran Companies | 0.84 | 21.52 | 36.53 | 20.68 | 15.01 |
| 9 | Attaqa | 0.69 | 17.28 | 33.82 | 16.59 | 16.54 |
| 10 | NIOF | 0.37 | 14.86 | 38.44 | 14.49 | 23.58 |
| 11 | Kazak Hassan | 0.95 | 14.48 | 35.85 | 13.53 | 21.37 |
| 12 | Attaka | 1.35 | 18.51 | 35.32 | 17.16 | 16.81 |
| 13 | Adabiya | 0.5 | 16.01 | 39.09 | 15.51 | 23.08 |
