# Supplementary material for: Mineralogical and chemical characterization of Suez Bay surface sediments via multi-analytical techniques
Source: Sci Rep. 2025 Oct 28;15:37729. doi: 10.1038/s41598-025-22518-w (PMC12568952; doi:10.1038/s41598-025-22518-w)
Supplement: Supplementary file 4 — Supplementary Material 4 [file 41598_2025_22518_MOESM4_ESM.docx]

**Mineralogical and Chemical Characterization of Suez Bay Surface Sediments via Multi-Analytical Techniques**

Randa R. Elmorsi^a^, Wael Abdel Wahhab^b^, Khaled S. Abou-El-Sherbini^,c^

*^a^ National Institute of Oceanography and Fisheries (NIOF), Egypt.*

*^b^ Geology Department, National Research Centre, 33 El Bohouth St. (former Tahrir St.), 12622, Dokki, Giza, Egypt.*

*^c^ Inorganic Chemistry Department, National Research Centre, 33 El Bohouth St. (former Tahrir St.), 12622, Dokki, Giza, Egypt.*

Supplementary Figure S1

Supplementary Figure S1 Continued XRF analysis of air-dried sediments
