# Supplementary material for: Mineralogical and chemical characterization of Suez Bay surface sediments via multi-analytical techniques
Source: Sci Rep. 2025 Oct 28;15:37729. doi: 10.1038/s41598-025-22518-w (PMC12568952; doi:10.1038/s41598-025-22518-w)
Supplement: Supplementary file 5 — Supplementary Material 5 [file 41598_2025_22518_MOESM5_ESM.zip › FTIR raw data/11.pdf]

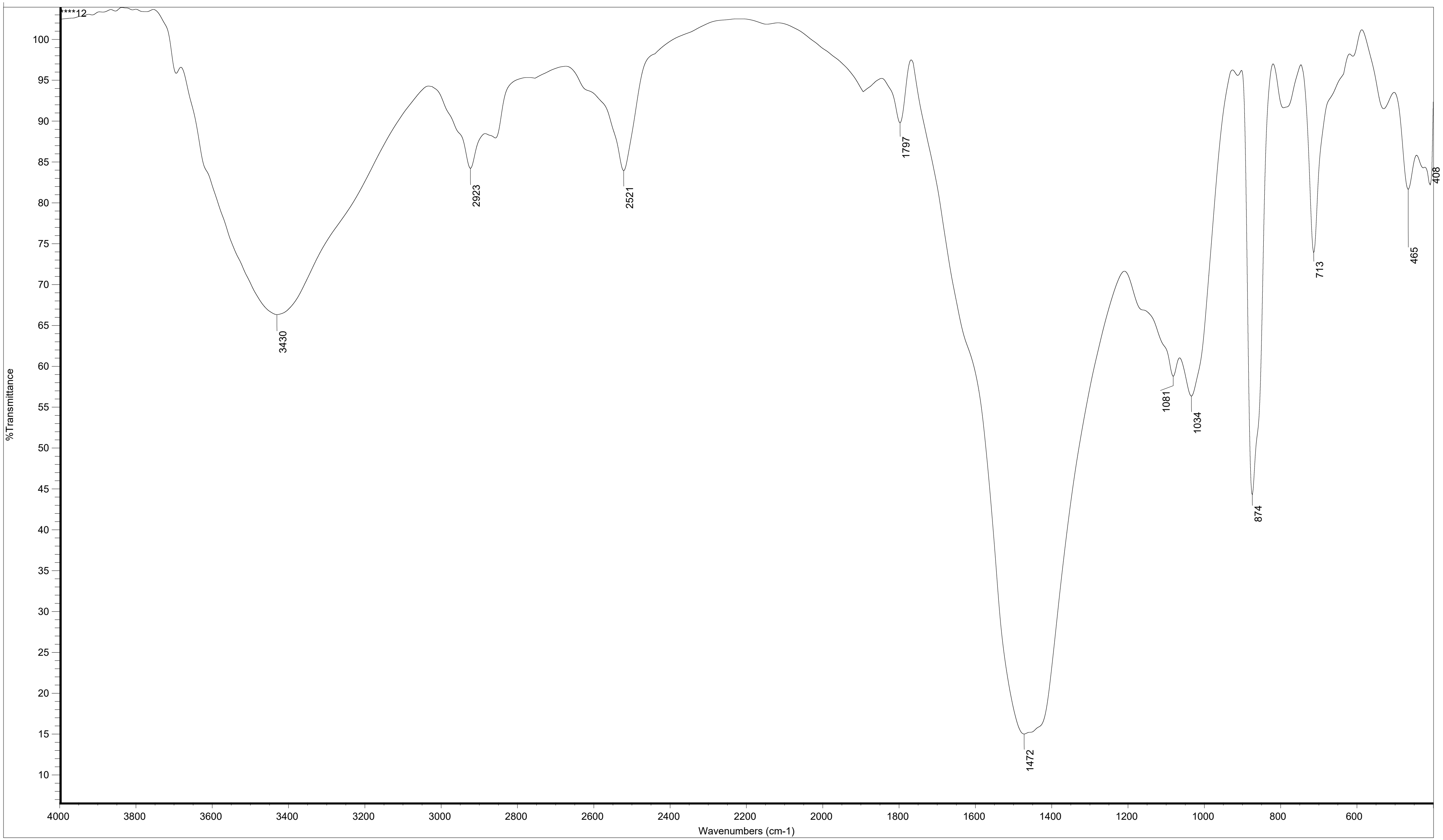


**Number of sample scans: 32**
**Number of background scans: 32**
**Resolution: 4.000**
**Sample gain: 4.0**
**Optical velocity: 0.4747**
**Aperture: 80.00**

ThermoFisher SCIENTIFIC

**Tue Aug 20 12:01:14 2019 (GMT+02:00)**


***Mansoura University***
***Faculty of Science***
***Spectral Analysis Unit***
***unitofspectra@gmail.com***
