# Supplementary material for: Mineralogical and chemical characterization of Suez Bay surface sediments via multi-analytical techniques
Source: Sci Rep. 2025 Oct 28;15:37729. doi: 10.1038/s41598-025-22518-w (PMC12568952; doi:10.1038/s41598-025-22518-w)
Supplement: Supplementary file 5 — Supplementary Material 5 [file 41598_2025_22518_MOESM5_ESM.zip › FTIR raw data/9.pdf]

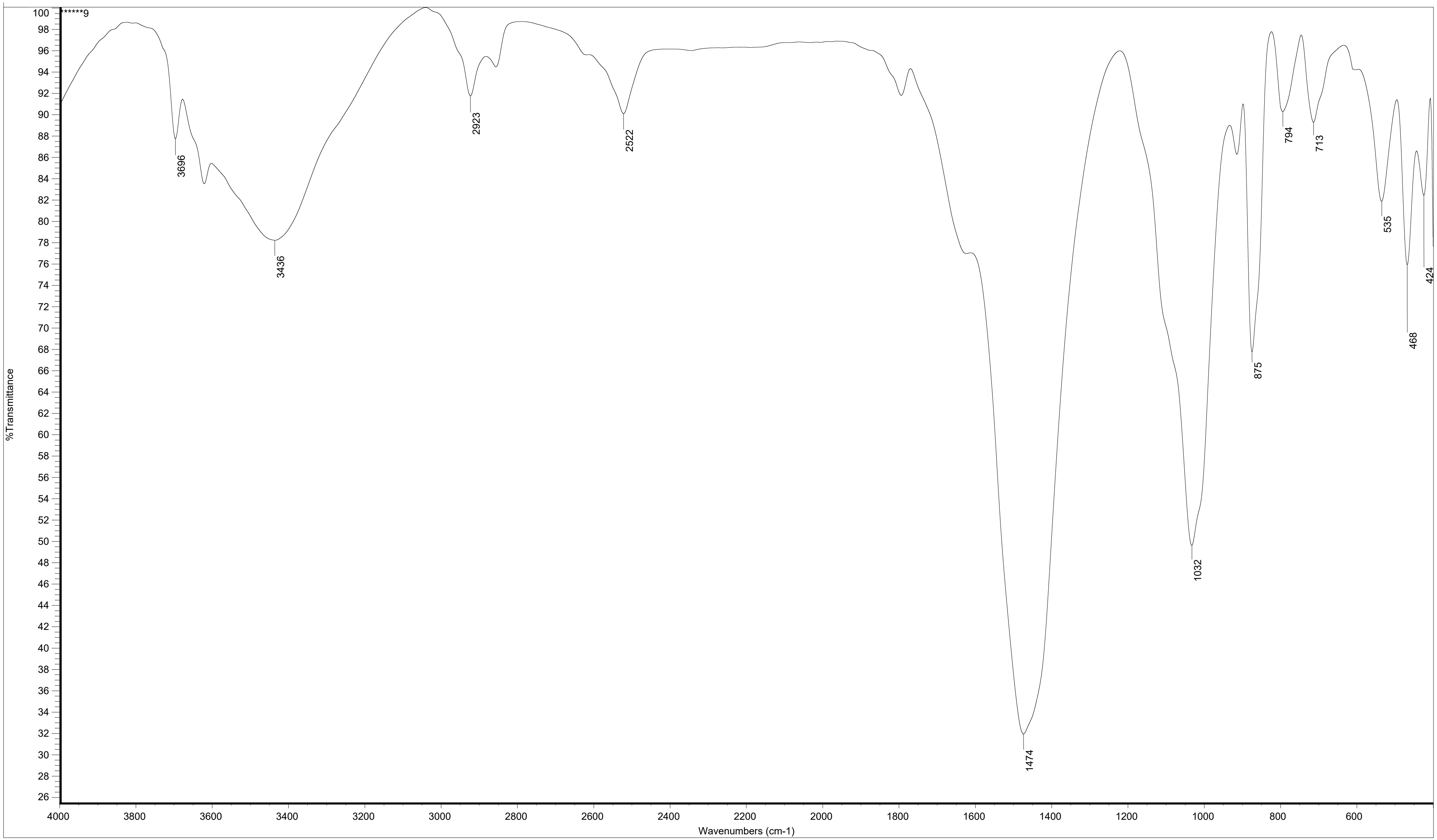

Number of sample scans: 32
Number of background scans: 32
Resolution: 4.000
Sample gain: 2.0
Optical velocity: 0.4747
Aperture: 80.00

Tue Aug 20 12:04:18 2019 (GMT+02:00)

Mansoura University
Faculty of Science
Spectral Analysis Unit
unitofspectra@gmail.com
