# Supplementary figures and images for: Mineralogical and chemical characterization of Suez Bay surface sediments via multi-analytical techniques
Source: Sci Rep. 2025 Oct 28;15:37729. doi: 10.1038/s41598-025-22518-w (PMC12568952; doi:10.1038/s41598-025-22518-w)

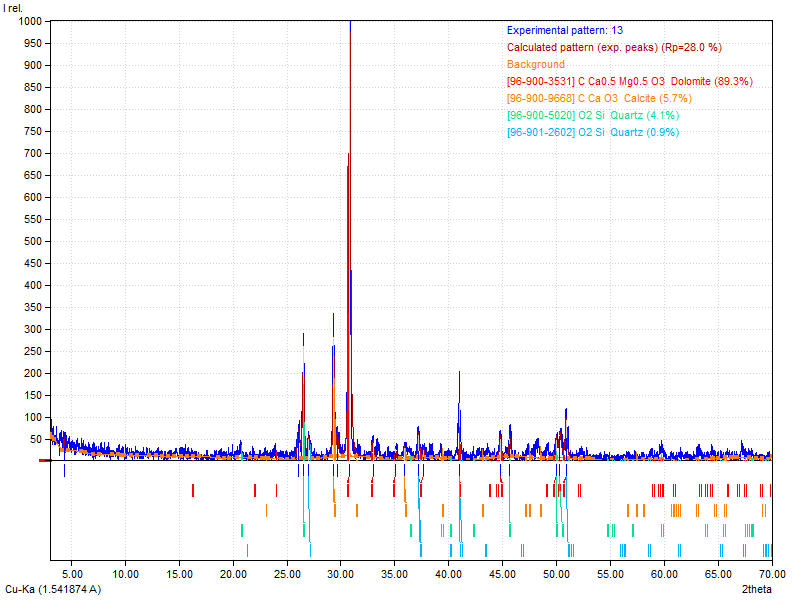

Supplement: Supplementary file 6 — Supplementary Material 6 [file 41598_2025_22518_MOESM6_ESM.zip › XRD raw data/13_Pattern.png]

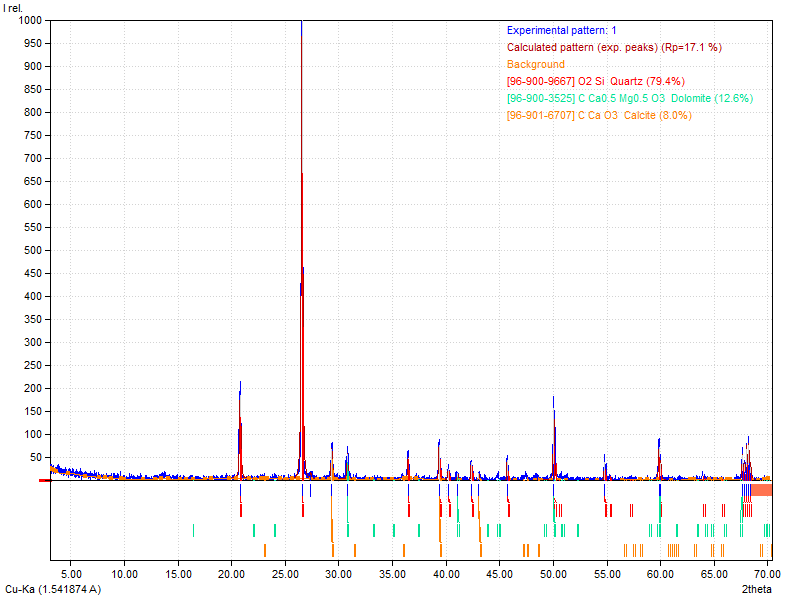

Supplement: Supplementary file 6 — Supplementary Material 6 [file 41598_2025_22518_MOESM6_ESM.zip › XRD raw data/1_Pattern.png]

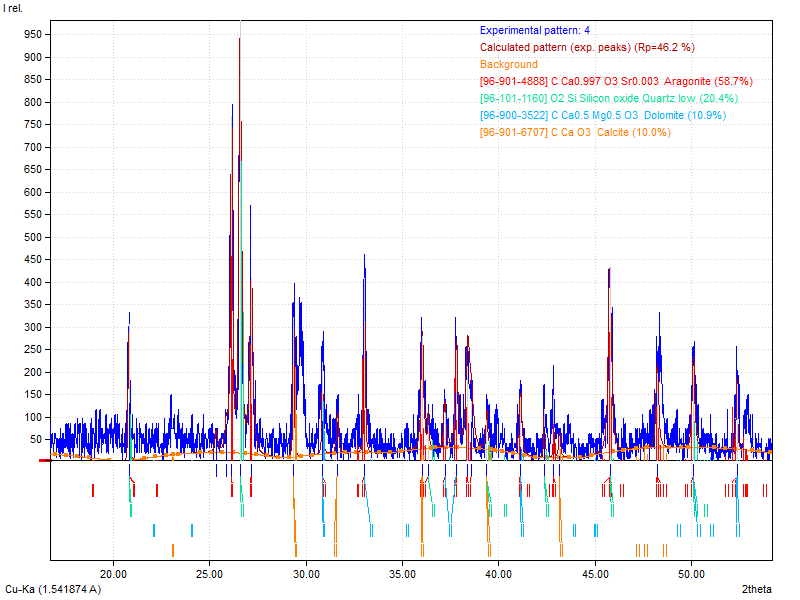

Supplement: Supplementary file 6 — Supplementary Material 6 [file 41598_2025_22518_MOESM6_ESM.zip › XRD raw data/4_Pattern.png]

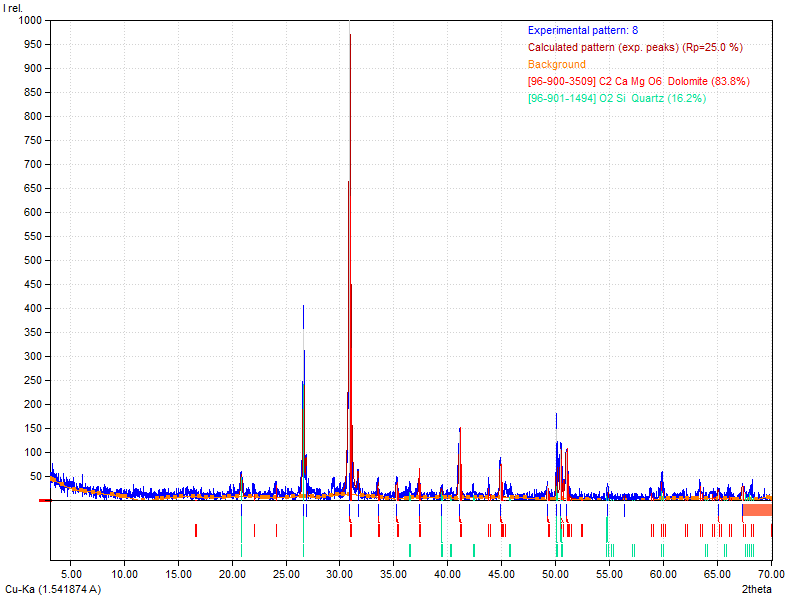

Supplement: Supplementary file 6 — Supplementary Material 6 [file 41598_2025_22518_MOESM6_ESM.zip › XRD raw data/8_Pattern.png]

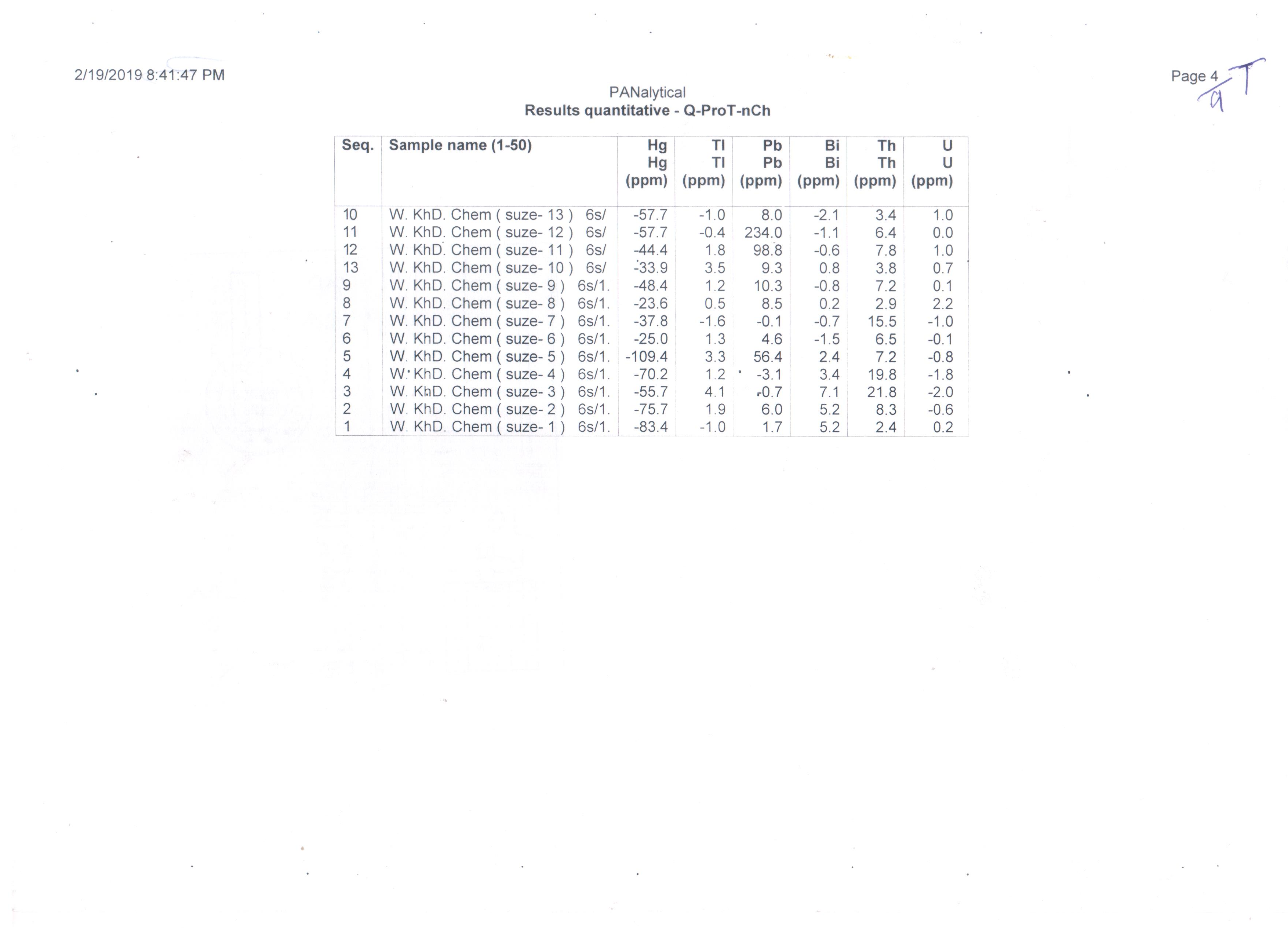

Supplement: Supplementary file 7 — Supplementary Material 7 [file 41598_2025_22518_MOESM7_ESM.zip › XRF raw data/Image (100).png]

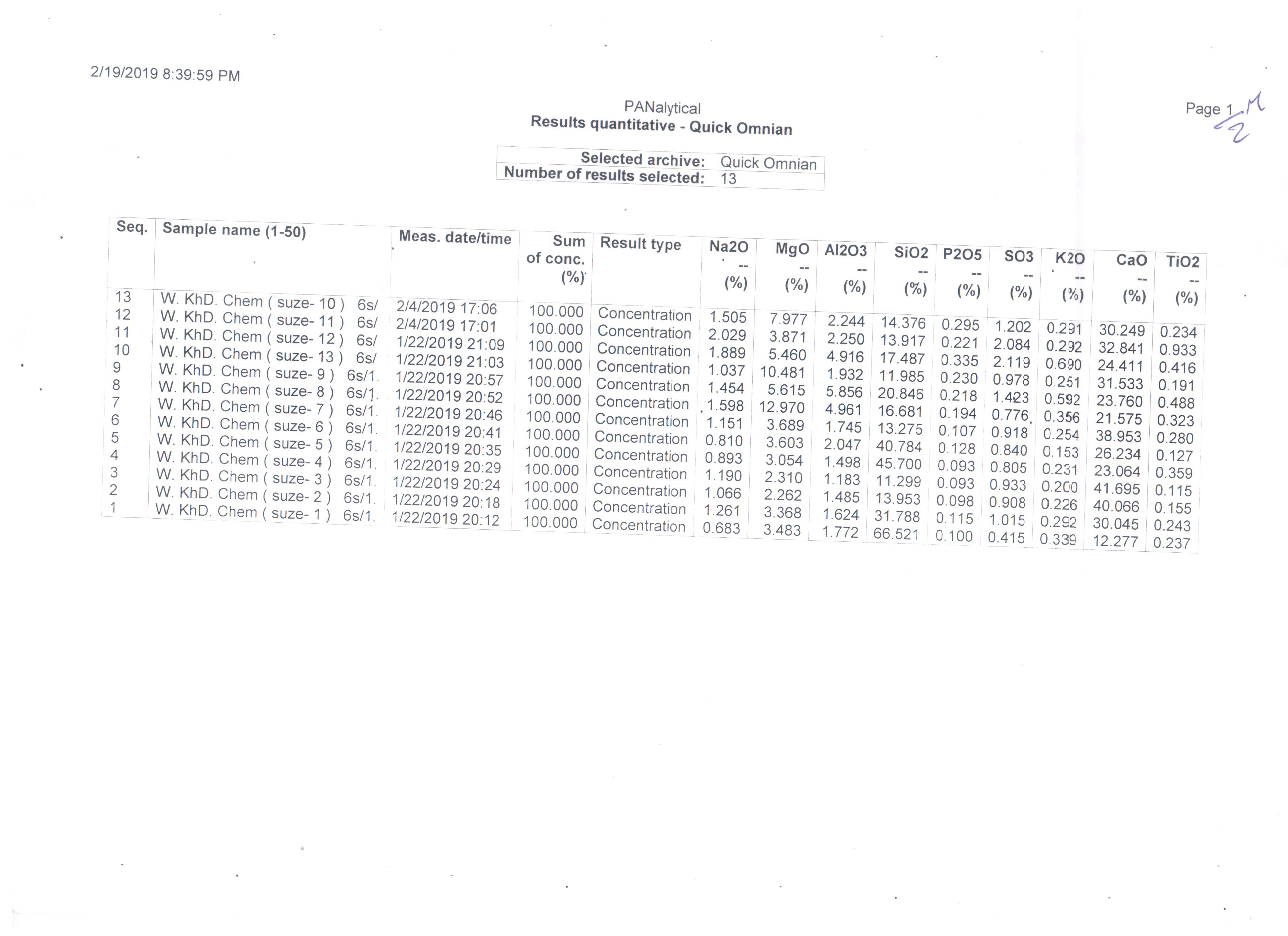

Supplement: Supplementary file 7 — Supplementary Material 7 [file 41598_2025_22518_MOESM7_ESM.zip › XRF raw data/Image (95).png]

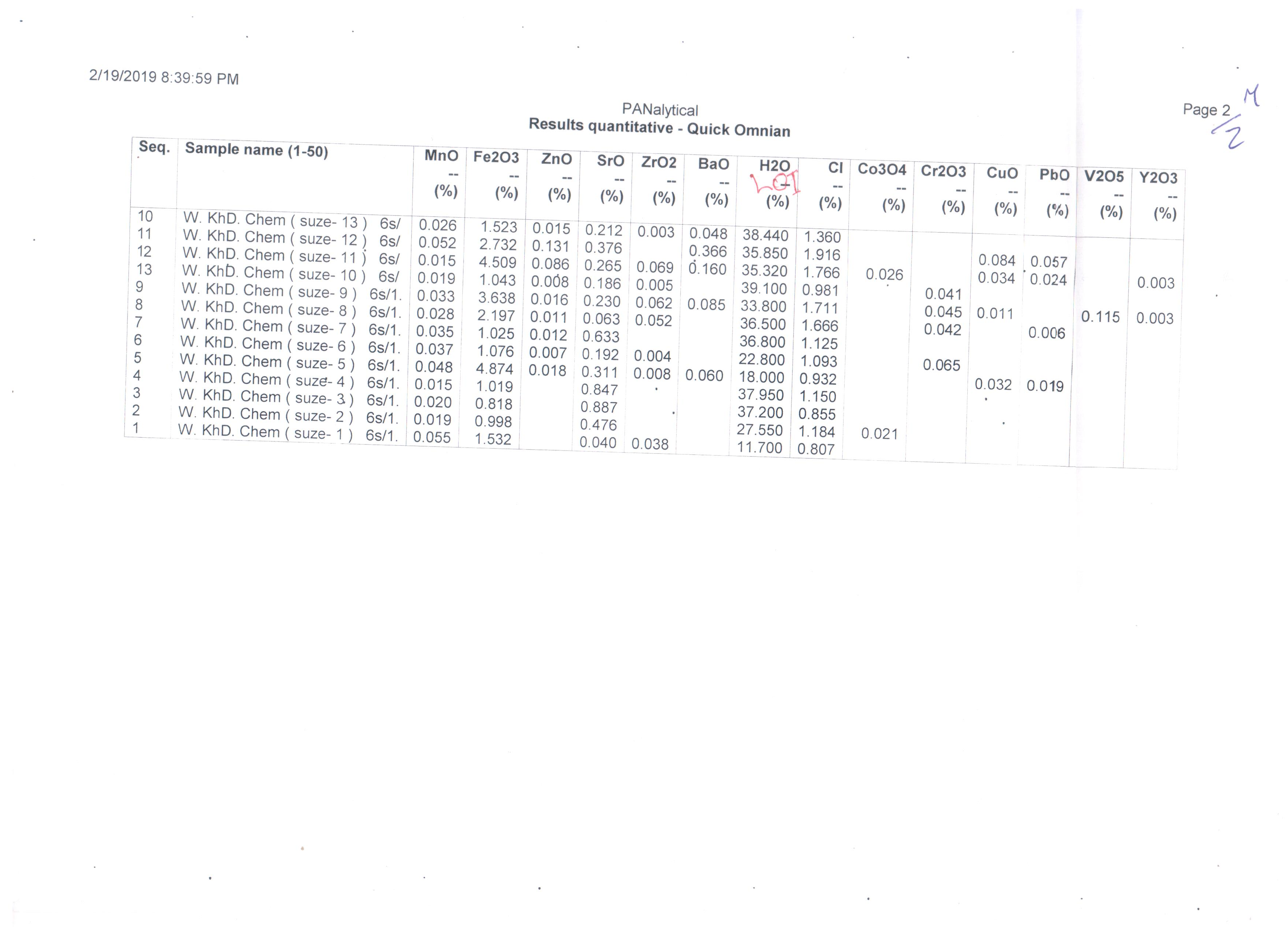

Supplement: Supplementary file 7 — Supplementary Material 7 [file 41598_2025_22518_MOESM7_ESM.zip › XRF raw data/Image (96).png]

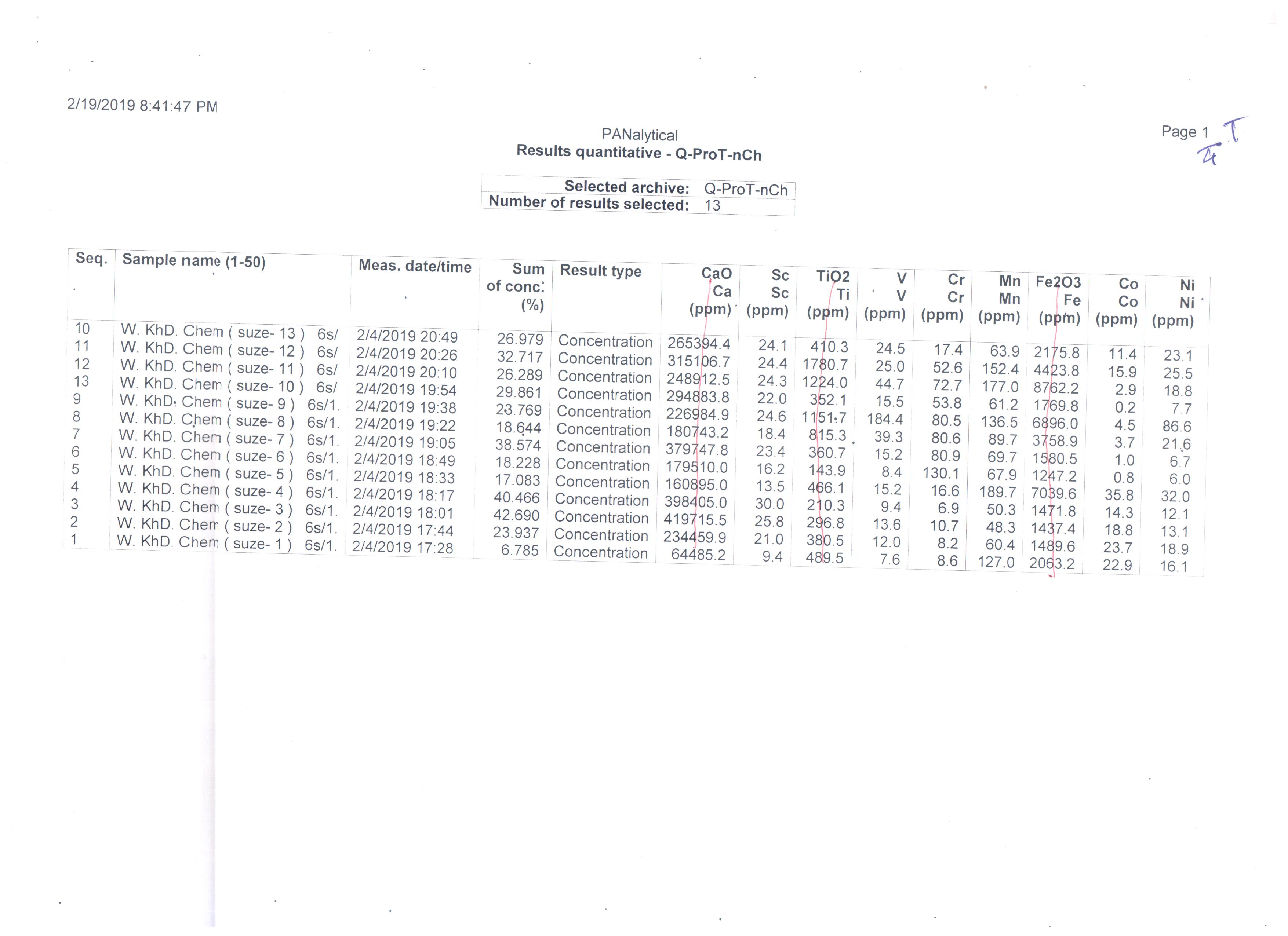

Supplement: Supplementary file 7 — Supplementary Material 7 [file 41598_2025_22518_MOESM7_ESM.zip › XRF raw data/Image (97).png]

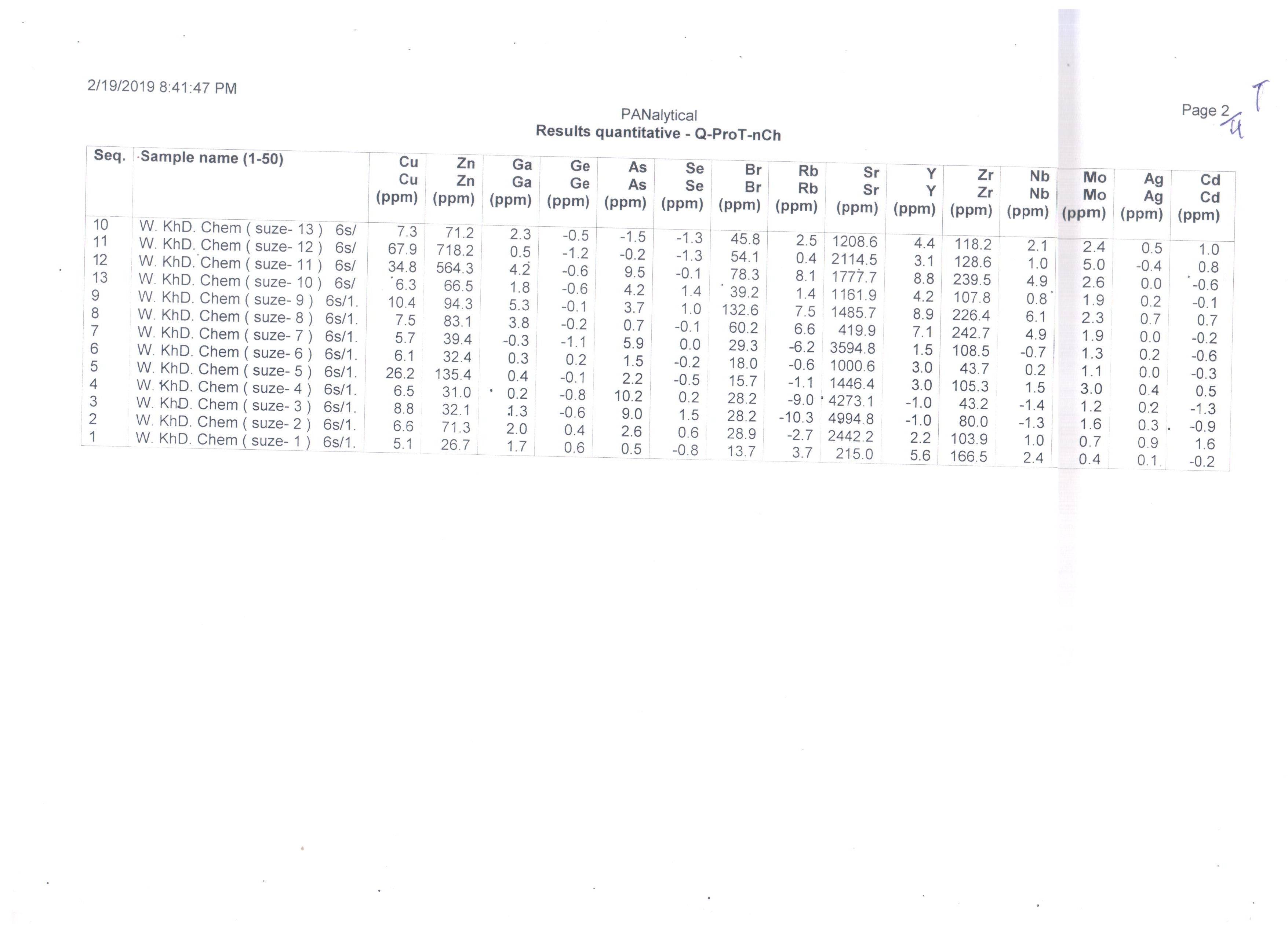

Supplement: Supplementary file 7 — Supplementary Material 7 [file 41598_2025_22518_MOESM7_ESM.zip › XRF raw data/Image (98).png]

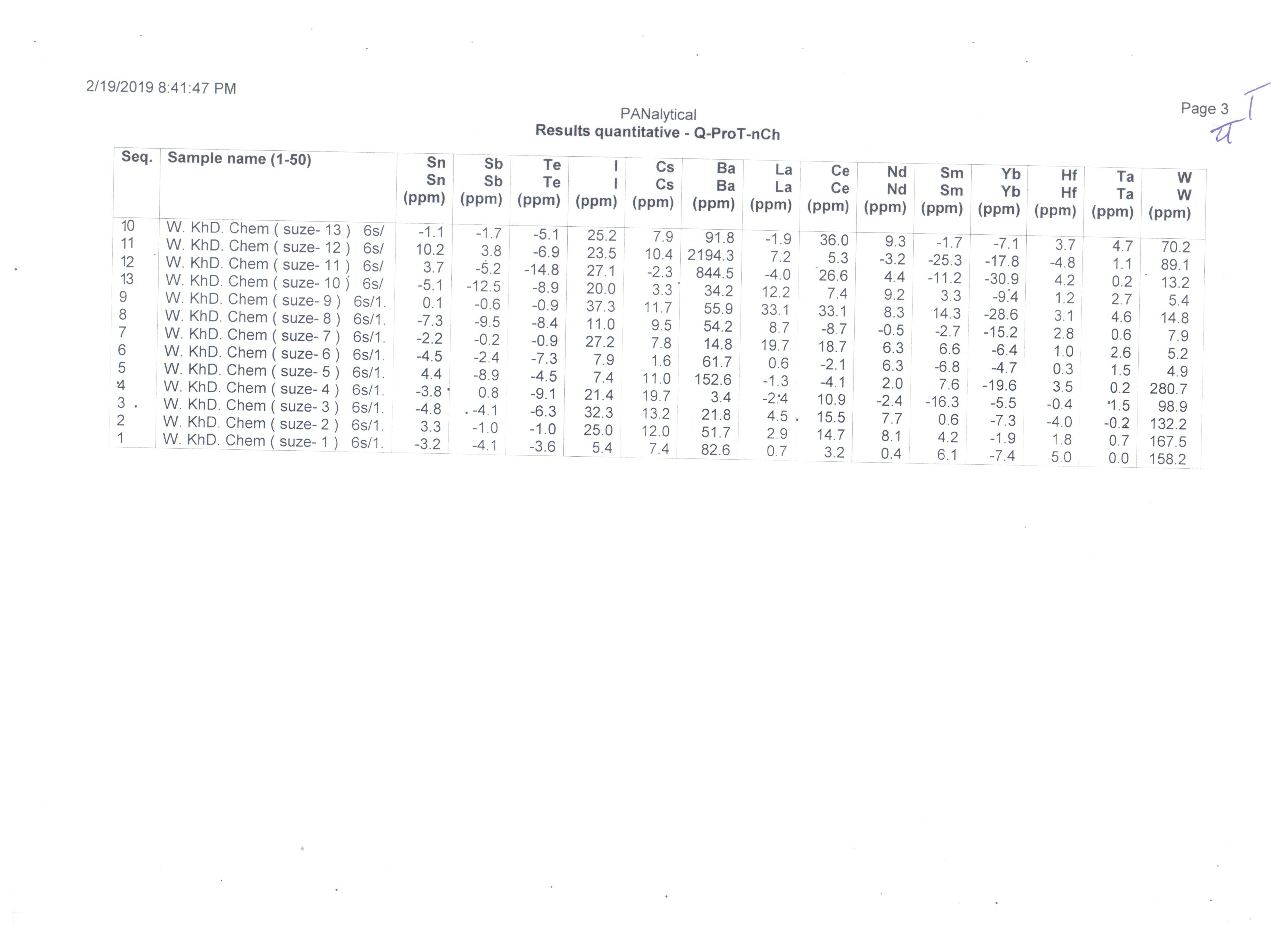

Supplement: Supplementary file 7 — Supplementary Material 7 [file 41598_2025_22518_MOESM7_ESM.zip › XRF raw data/Image (99).png]
