# Supplementary material for: Mineralogical and chemical characterization of Suez Bay surface sediments via multi-analytical techniques
Source: Sci Rep. 2025 Oct 28;15:37729. doi: 10.1038/s41598-025-22518-w (PMC12568952; doi:10.1038/s41598-025-22518-w)
Supplement: Supplementary file 8 — Supplementary Material 8 [file 41598_2025_22518_MOESM8_ESM.docx]

**Mineralogical and Chemical Characterization of Suez Bay Surface Sediments via Multi-Analytical Techniques**

Randa R. Elmorsi^a^, Wael Abdel Wahhab^b^, Khaled S. Abou-El-Sherbini^,c^

*^a^ National Institute of Oceanography and Fisheries (NIOF), Egypt.*

*^b^ Geology Department, National Research Centre, 33 El Bohouth St. (former Tahrir St.), 12622, Dokki, Giza, Egypt.*

*^c^ Inorganic Chemistry Department, National Research Centre, 33 El Bohouth St. (former Tahrir St.), 12622, Dokki, Giza, Egypt.*

Table S2 FTIR spectra of sediment samples from selected sites (8-13)

| Type of vibration | Wave number, cm^-1^ | | | | | | Reference |
| --- | --- | --- | --- | --- | --- | --- | --- |
|  | Site 8 | Site 9 | Site 10 | Site 11 | Site 12 | Site 13 |  |
| tetrahedral δ_Si-O-Si_ | 423.3 | 420.4 | 421.4 | 422.3 | 425.2 | 431.0 | [1] |
| Tetrahedral δ_Si-O-Mg_ | 466.7 | 467.7 | 465.7 | 466.7 | 466.7 | 465.7 | [1] |
| Tetrahedral δ_Si-O-Al_ | 530.3 | 529.4 | 534.2 | 530.3 | 530.3 | 537.1 | [1] |
| Kaolin | 692.3 | 690.4 | 692.3 | 693.3 | 692.3 | 693.3 | [2] |
| deformation vibration of hydroxyls in [Fe(III)Fe(III)-OH] and [Fe(III)Mg-OH] | 793.6 | 792.6 | 793.6 | 793.6 | 792.6 | 794.5 | [2] |
| smectite 2:1 deformation Al-Al-OH | 914.9 | 915.1 | 913.1 | 914.1 | 914.9 | 914.1 | [1] |
| ν_SiO_ | 1032.7 | 1033.7 | 1032.7 | 1032.7 | 1033.0 | 1033.7 | [1] |
| νCO_3_ | 1384.6 | - | 1382.7 | 1403.9 | - | 1386.6 | [1] |
| νCO_3_ | 1464.7 | 1430.9 | - | - | - | 1458.9 | [1] |
| δ_OH_ | 1643.1 | 1640.2 | 1643.1 | 1642.1 | 1643.1 | 1639.2 | [1] |
| ν_CH_ | 2923 | 2923 | 2924 | 2923 | 2922 | 2923 | [1] |
| ν_OH_ | 3442.3 | 3444.2 | 3439.4 | 3429.8 | 3421.1 | 3431.7 | [1] |
| ν_Al-OH_ | 3623.6 | 3624.6 | 3622.6 | 3623.6 | 3624.6 | 3620.7 | [2] |
| ν_Al-OH_ in kaolinite | 3696.9 | 3696.9 | 3696.9 | 3696.9 | 3696.9 | 3694.0 | [2] |

References

[1] Russell, D. & Fraser, A. R. Clay Mineralogy : Spectroscopic and Chemical Determinative Methods. in (ed. Wilson, M. J.) (Chapman & HalI, Pondicherry, 1994).

[2] Letaïef, S., Casal, B., Aranda, P., Martín-Luengo, M. A. & Ruiz-Hitzky, E. Fe-containing pillared clays as catalysts for phenol hydroxylation. Appl Clay Sci 22, 263–277 (2003).
